# Supplementary material for: Externally-triggerable optical pump-probe scanning tunneling microscopy with a time resolution of tens-picosecond
Source: Sci Rep. 2023 Jan 25;13:818. doi: 10.1038/s41598-023-27383-z (PMC9877009; doi:10.1038/s41598-023-27383-z)
Supplement: Supplementary file 1 — Supplementary Information. [file 41598_2023_27383_MOESM1_ESM.pdf]

Supplementary Information

Externally-triggerable optical pump-probe scanning tunneling microscopy with a time resolution of tens-picosecond

Katsuya Iwaya[1], Munenori Yokota[1], Hiroaki Hanada[1], Hiroyuki Mogi[2], Shoji Yoshida[2], Osamu Takeuchi[2], Yutaka Miyatake[1], and Hidemi Shigekawa[2],

[1]UNISOKU Co., Ltd., Hirakata, Osaka 573-0131, Japan
[2]Faculty of Pure and Applied Sciences, University of Tsukuba, Tsukuba, Ibaraki 305-8573, Japan

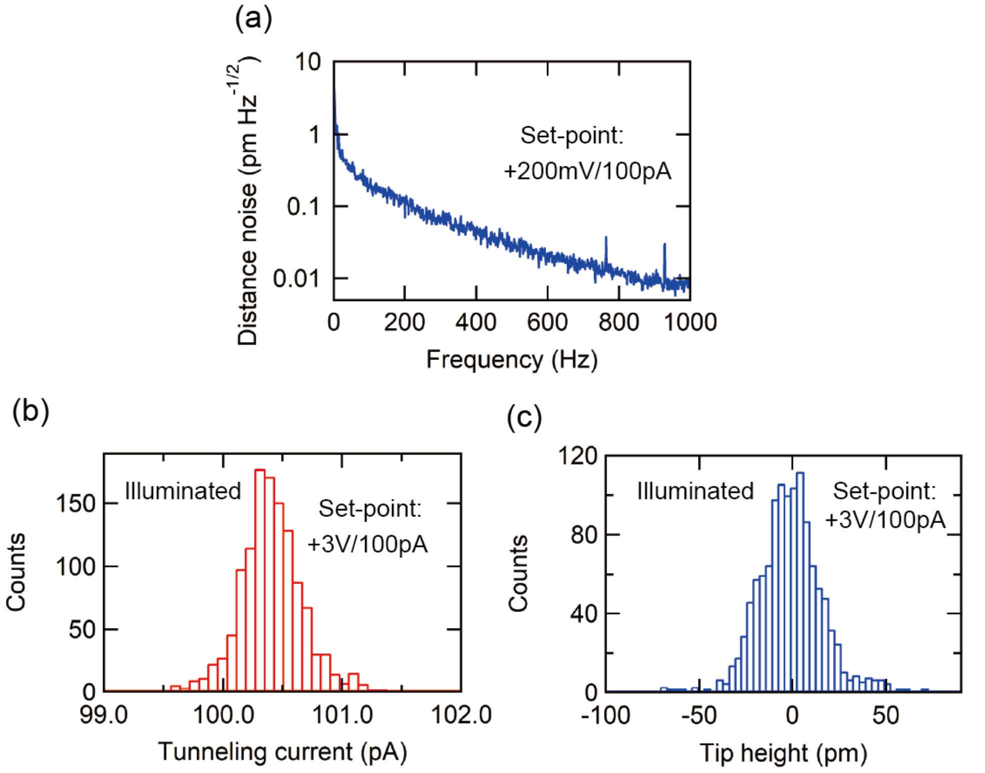

Figure S1. (a) Typical tip-sample distance noise spectrum taken when the feedback loop closed without laser illumination. T = 78 K. The sample is highly ordered pyrolytic graphite (HOPG). Set-point: V = +200 mV, I_t = 100 pA. (b, c) Histogram of the tunneling current and the tip height with laser illumination on GaAs(110) surface for 12 hours, respectively. T = 6 K. Set-point: V = +3 V, I_t = 100 pA. Laser power is 0.25 mW. The tunneling current and tip height were measured every 30 seconds. The average tunneling current (the tip height) and the standard deviation of tunneling current (tip height) are 100.4 pA (-2.2 pm) and 0.3 pA (20.1 pm), respectively.

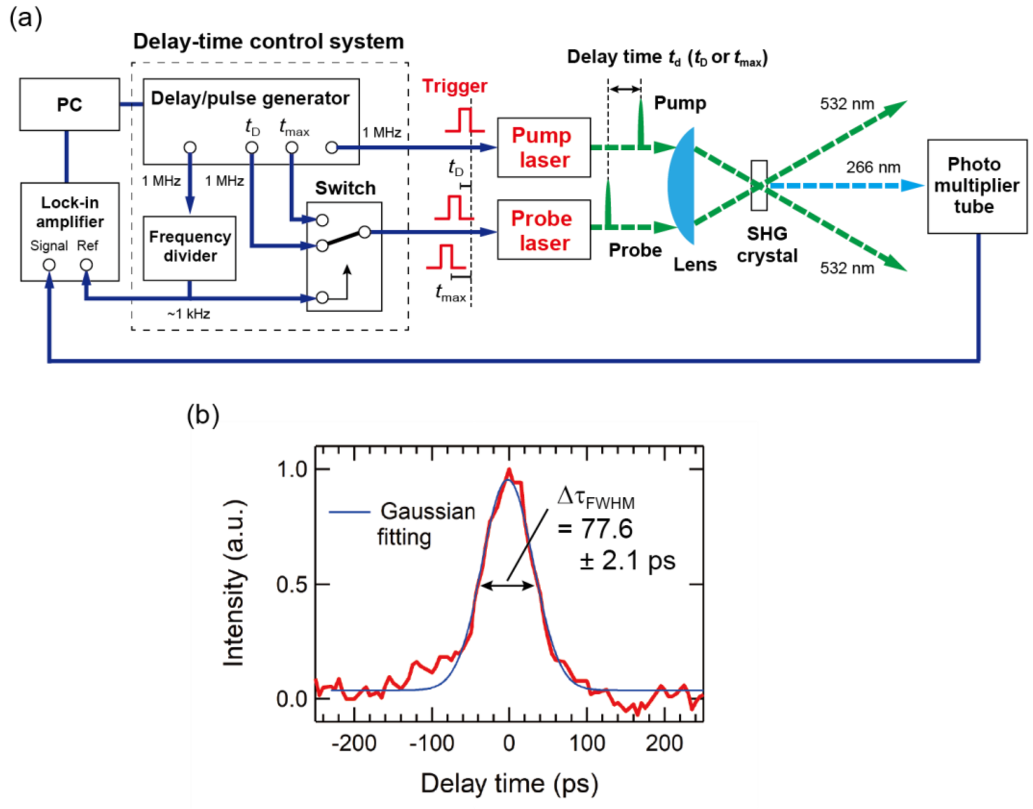

Figure S2. Cross-correlation measurement between pump and probe laser pulses using the sum frequency generation. (a) Schematic diagram of the system. SHG: second-harmonic generation. (b) Cross-correlation as a function of delay time. The correlation width estimated by Gaussian fitting is 77.6 ± 2.1 ps.
